# Can neoadjuvant chemotherapy improve survival in stage T3-4N1 nasopharyngeal carcinoma? A propensity matched analysis

**DOI:** 10.1186/s13014-020-01594-4

**Published:** 2020-07-02

**Authors:** Lei Wang, Zheng Wu, Dehuan Xie, Shaowen Lv, Liangping Xia, Yong Su

**Affiliations:** 1 VIP Region, Sun Yat-sen University Cancer Center, State Key Laboratory of Oncology in South China, Collaborative Innovation Center for Cancer Medicine, No. 651 Dongfeng Road East, Guangzhou, 510060 People’s Republic of China; 2 Department of Radiation Oncology, Hunan Cancer Hospital and The Affiliated Cancer Hospital of Xiangya School of Medicine, Central South University, 283 Tong Zi Po Road, Changsha, 410013 People’s Republic of China; 3 Department of Radiation Nasopharyngeal Carcinoma, Sun Yat-sen University Cancer Center, State Key Laboratory of Oncology in South China, Collaborative Innovation Center for Cancer Medicine, No. 651 Dongfeng Road East, Guangzhou, 510060 People’s Republic of China; 4 Department of Radiation Oncology, Sun Yat-sen University Cancer Center, State Key Laboratory of Oncology in South China, Collaborative Innovation Center for Cancer Medicine, No. 651 Dongfeng Road East, Guangzhou, 510060 People’s Republic of China

**Keywords:** Nasopharyngeal neoplasm, Neoadjuvant chemotherapy, Intensity-modulated radiotherapy, Prognosis

## Abstract

**Background:**

To estimate the efficacy of neoadjuvant chemotherapy (NCT) in stage T3-4N1 nasopharyngeal carcinoma (NPC).

**Methods:**

Data on stage T3-4N1 NPC patients treated with concurrent chemoradiotherapy (CCRT) with or without NCT at the Sun Yat-sen University Cancer Center between January 2006 and December 2013 were retrospectively reviewed. Propensity score matching (PSM) was carried out to balance prognostic factors in NCT followed by CCRT (NCT + CCRT) group and CCRT group in a 1:1 ratio. Survival outcomes of matched patients in the two groups were compared, and prognostic factors were identified using Cox regression model.

**Results:**

A total of 282 patients were involved in this study, with 136 of NCT + CCRT group and 146 of CCRT group. After PSM, 85 pairs of patients were selected. There were no significant differences in 5-year overall survival (OS), locoregional recurrence-free survival (LRFS), distant recurrence-free survival (DRFS), and recurrence-free survival (RFS) between NCT + CCRT group and CCRT group (81.0% vs. 77.5%, *P* = 0.750; 85.8% vs. 88.1%, *P* = 0.495; 92.5% vs. 93.9%, *P* = 0.759; 81.0% vs.77.5%, *P* = 0.919, respectively). Multivariate analysis found that smoking history (*P* = 0.044) and T classification (*P* = 0.027) were independent prognostic factors for OS, lymph node diameter (*P* = 0.032) was independent prognostic factor for LRFS, positive pretreatment lymph node condition (PLNC), which was defined as the lymph node necrosis or confluent, was independent prognostic factor for DRFS (*P* = 0.007), and RFS (*P* = 0.009). Lower 5-year OS (82.7% vs. 94.1%, *P* = 0.014), DRFS (79.3% vs. 96.2%, *P* = 0.003), and RFS (62.4% vs. 86.8%, *P* = 0.001) were found in positive PLNC group compared with negative PLNC group. In terms of toxicities, the incidences of acute hematological Grade 3–4 adverse events (AEs) were higher in NCT + CCRT group compared with CCRT group (*P* < 0.05), while no significant difference was observed in the rates of non-hematological Grade 3–4 AEs between these two groups (*P* > 0.05).

**Conclusions:**

Additional NCT is not associated with improved survival outcomes for patients with stage T3-4N1 NPC, but bring increased hematological Grade 3–4 AEs. PLNC is independent prognostic factor in stage T3-4N1 NPC, with positive PLNC correlating with poor survival outcomes.

## Background

Nasopharyngeal carcinoma (NPC) is a highly chemoradiosensitive tumor with specific geographic distribution [1]. More than 70% of patients are diagnosed with locoregionally advanced disease at presentation [2] with unfavorable prognosis receiving concurrent chemoradiotherapy (CCRT), which is the predominant treatment modality for locoregionally advanced NPC (LANPC) [3]. With the application of intensity-modulated therapy (IMRT), higher dose to the target and better protection of organs at risk (OARs) are available compared to two-dimentional conventional radiotherapy (2DCRT) [4]. Better locoregional control is observed in IMRT era [5], and distant metastasis becomes the main treatment failure [6]. Therefore, neoadjuvant chemotherapy (NCT) has been applied greatly in order to improve distant control in LANPC. Recent prospective trials investigating the efficacy of NCT in LANPC demonstrated improved survival outcomes [7–9]. However, the N1 stage disease has lower risk of distant metastasis compared with N2–3 disease [10]. Thus, the benefit of NCT in LANPC with N1 disease remains investigational. Based on this premise, we conducted this retrospective study to clarify the value of NCT in patients with stage T3-4N1 NPC, aiming to provide clinicians with reference of individualized treatment choices.

## Materials and methods

### Patients

Newly diagnosed nonkeratinizing NPC with stage T3-4N1 disease according to American Joint Committee on Cancer/ Union for International Cancer Control (AJCC/ UICC) 8th edition between January 2006 and December 2013 at the Sun Yat-sen University Cancer Center using IMRT were reviewed. Exclusion criteria were as follows: (1) Karnofsky performance score < 80; (2) age of < 18 or > 75 years old; (3) distant metastasis at diagnosis; (4) a history of cancer within 5 years, (5) receipt of previous treatment to the nasopharynx or neck; (6) receipt of NCT less than two cycles; (7) receipt of adjuvant chemotherapy or target therapy, (8) no concurrent chemotherapy (CCT); (9) lactation or pregnancy. All clinical records and pretreatment magnetic resonance imaging (MRI) materials were reviewed. The pretreatment lymph node condition (PLNC) was evaluated: the lymph node necrosis or confluent was classified as positive, while non-necrosis or non-confluent was classified as negative. The study was approved by the Medical Ethics Committee of Sun Yat-sen University Cancer Center, and the need for written informed consent was waived. Key data of this study has been uploaded onto the Research Data Deposit public platform (http://www.researchdata.org.cn), with approval number of RDDA2020001460.

### Radiotherapy

The details of IMRT have been previously reported. Target volumes and OARs were determined in accordance with the International Commission on Radiation Units and Measurements Reports (ICRU) 50 and 62 as well as our institutional treatment protocol [11]. Gross tumor volume (GTV) included GTVp defining as the primary gross tumor (including retropharyngeal lymph node metastases), and GTVnd defining as cervical lymph node metastasis. The high-risk clinical target volume (CTV1) was defined as GTVp plus a 5–10 mm margin and the whole nasopharynx, and the low-risk clinical target volume (CTV2) was defined as CTV1 plus a 5–10 mm margin together with the bilateral cervical selective lymph drainage areas. The prescribed doses were 66–72 Gy, 64–70 Gy, 60–62 Gy, and 50–54 Gy, in 28–32 fractions, for the planning target volume (PTV) derived from GTVp, GTVnd, CTV1, and CTV2, respectively.

### Chemotherapy

The platinum based NCT regimens included (1) PF regimen: cisplatin (80 mg/m2) plus 5-fluorouracil (800 mg/m2/day over 120 h); (2) TP regimen: cisplatin (80 mg/m2) plus docetaxel (80 mg/m2); (3) TPF regimen: cisplatin (60 mg/m2) plus 5-fluorouracil (600 mg/m2 over 120 h) with docetaxel (60 mg/m2). All NCT regimens were administered at 3-week interval. The CCT regimen was cisplatin (80–100 mg/m2) every 3 weeks for 2–3 cycles. Toxicities during treatment were assessed based on the Common Terminology Criteria for Adverse Events (version 3.0).

### Endpoints and follow-up

Follow-up was measured from the first day of therapy to last examination or death. In the first 3 years, patients were assessed every 3 months, and then every 6–12 months thereafter until death or loss of follow-up. The endpoints were overall survival (OS), locoregional recurrence-free survival (LRFS), distant recurrence-free survival (DRFS), and recurrence-free survival (RFS), which were defined as the time from treatment to death for any cause; to locoregioanl recurrence; to distant recurrence; and to locoregional recurrence, distant recurrence or death for any cause, respectively.

### Statistical methods

The Mann-Whitney U test was used for ordinal variables, and the chi-squared test was used for nominal variables. A propensity score matching (PSM) method was performed to match patients from NCT + CCRT group and CCRT group in a 1:1 ratio using the following covariates: sex, age, smoking history, alcohol history, family of cancer, T classification, PLNC, lymph node diameter, cervical lymph node, pretreatment EBV DNA copy. The survival outcomes and differences were evaluated using the Kaplan-Meier method and the log-rank test. The Cox regression model was used to identify the independent prognostic factors in parameters of covariates illustrated above in PSM method as well as pretreatment hemoglobin (HGB), C-reactive protein (CRP), and lactate dehydrogenase (LDH). The criterion for statistical significance was set at α = 0.05. *P*-values were determined from two-sided tests. All analyses were carried out with the SPSS 21.0 software (SPSS Inc., Chicago, IL, USA).

## Results

### Patient characteristics

**Fig. 1:**
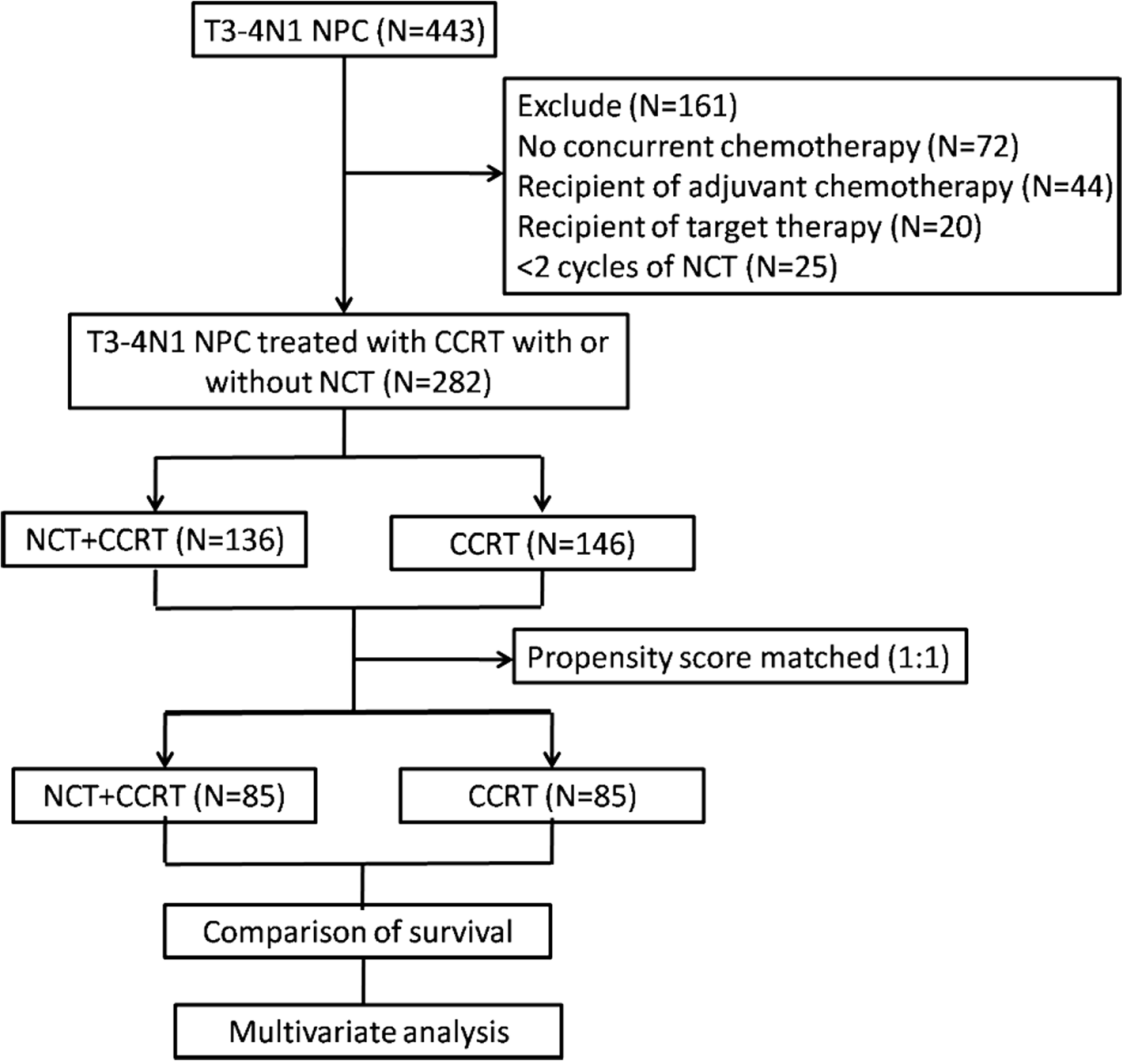
Flowchart of patients. NPC, nasopharyngeal carcinoma; NCT, neoadjuvant chemotherapy; CCRT, concurrent chemoradiotherapy

A total of 282 stage T3-4N1 NPC patients treated with or without NCT were involved (Fig. 1). The T classification (*P* < 0.001) and EBV DNA (*P* = 0.013) were not well balanced between NCT + CCRT group and CCRT group. After propensity score matching between two groups in 1:1 ratio, 85 pairs of patients were selected for further assessment (Table 1). Of the selected 170 patients, the median age was 43 years old (range, 22–73 years old), and 123/170 patients were male. The MRI materials before treatment were involved, with the median lymph node diameter of 14.0 mm (range, 4–41 mm). 18 (21.2%) patients had positive PLNC in NCT + CCRT group, while 15 (17.6%) patients had positive PLNC in CCRT group. Besides, there were 63 (74.1%) and 59 (69.4%) patients of cervical lymph node metastasis in NCT + CCRT group and CCRT group, respectively. Moreover, more than half of the matched cohort had Epstein-Barr virus (EBV) DNA > 2000 copy/ml.

**Table 1:** Baseline characteristics of patients with stage T3-4N1 NPC before and after match

**Variable**	**Before match**			**After match**		
	**NCT followed by CCRT group (136)**	**CCRT group (146)**	***P*value**	**NCT followed by CCRT group (85)**	**CCRT group** **(85)**	***P*value**
Sex			0.927			0.644
Male	103 (75.7%)	111 (76.0%)		63 (74.1%)	60 (70.6%)	
Female	32 (23.5%)	35 (24.0%)		21 (24.7%)	25 (29.4%)	
Age (years)	Median: 45, range: 21–73	Median: 45, range: 23–73	0.795	Median: 41, range: 22–73	Median: 44, range: 23–66	0.287
Smoking history			0.660			0.858
No	88 (64.7%)	98 (67.1%)		64 (75.3%)	65 (76.5%)	
Yes	48 (35.3%)	48 (32.9%)		21 (24.7%)	20 (23.5%)	
Alcohol history			0.181			0.073
No	117 (86.0%)	133 (91.1%)		76 (89.4%)	82 (96.5%)	
Yes	19 (14.0%)	13 (8.9%)		9 (10.6%)	3 (3.5%)	
Family of cancer			0.177			0.093
No	103 (75.7%)	100 (68.5%)		65 (76.5%)	55 (64.7%)	
Yes	33 (24.3%)	46 (31.5%)		20 (23.5%)	30 (35.3%)	
T classification			< 0.001			1.000
T3	67 (49.3%)	128 (87.7%)		67 (78.8%)	67 (78.8%)	
T4	69 (50.7%)	18 (12.3%)		18 (21.2%)	18 (21.2%)	
Lymph node diameter (mm, maximum)	Median: 14.5, range: 4–36	Median: 14, range: 4–41	0.155	Median: 16, range: 5–36	Median: 12, range: 4–41	0.138
PLNC			0.181			
Negative	104 (76.5%)	121 (82.9%)		67 (78.8%)	70 (82.4%)	
Positive	32 (23.5%)	25 (17.1%)		18 (21.2%)	15 (17.6%)	0.562
Cervical lymph node			0.442			0.497
No	41 (30.1%)	38 (26.0%)		22 (25.9%)	26 (30.6%)	
Yes	95 (69.9%)	108 (74.0%)		63 (74.1%)	59 (69.4%)	
EBV DNA (copy/ml)			0.013			1.000
≤ 2000	59 (43.4%)	85 (58.2%)		39 (45.9%)	39 (45.9%)	
> 2000	77 (56.6%)	61 (41.8%)		46 (54.1%)	46 (54.1%)	
HGB, g/L			0.918			0.683
< 113	3 (2.2%)	1 (0.7%)		3 (3.5%)	1 (1.2%)	
113–151	87 (64.0%)	98 (67.1%)		51 (60.0%)	57 (67.1%)	
≥ 151	46 (33.8%)	47 (32.2%)		31 (36.5%)	27 (31.8%)	
CRP, g/ml			0.057			0.104
< 1.0	45 (33.1%)	59 (40.4%)		28 (32.9%)	39 (45.9%)	
1.0–3.0	44 (32.4%)	53 (36.3%)		30 (35.3%)	25 (29.4%)	
≥ 3.0	47 (34.6%)	34 (23.3%)		27 (31.8%)	21 (24.7%)	
LDH, U/L			0.349			0.756
< 245	127 (93.4%)	140 (95.9%)		79 (92.9%)	80 (94.1%)	
≥ 245	9 (6.6%)	6 (4.1%)		6 (7.1%)	5 (5.9%)	

Abbreviations: *NPC* Nasopharyngeal carcinoma; *NCT* Neoadjuvant chemoradiotherapy; *CCRT* Concurrent chemoradiotherapy, *PLNC* Pretreatment lymph node condition; *EBV* Epstein–Barr virus; *HGB* Hemoglobin; *CRP* C-reactive protein; *LDH* Lactate dehydrogenase

### Survival outcomes

**Fig. 2:**
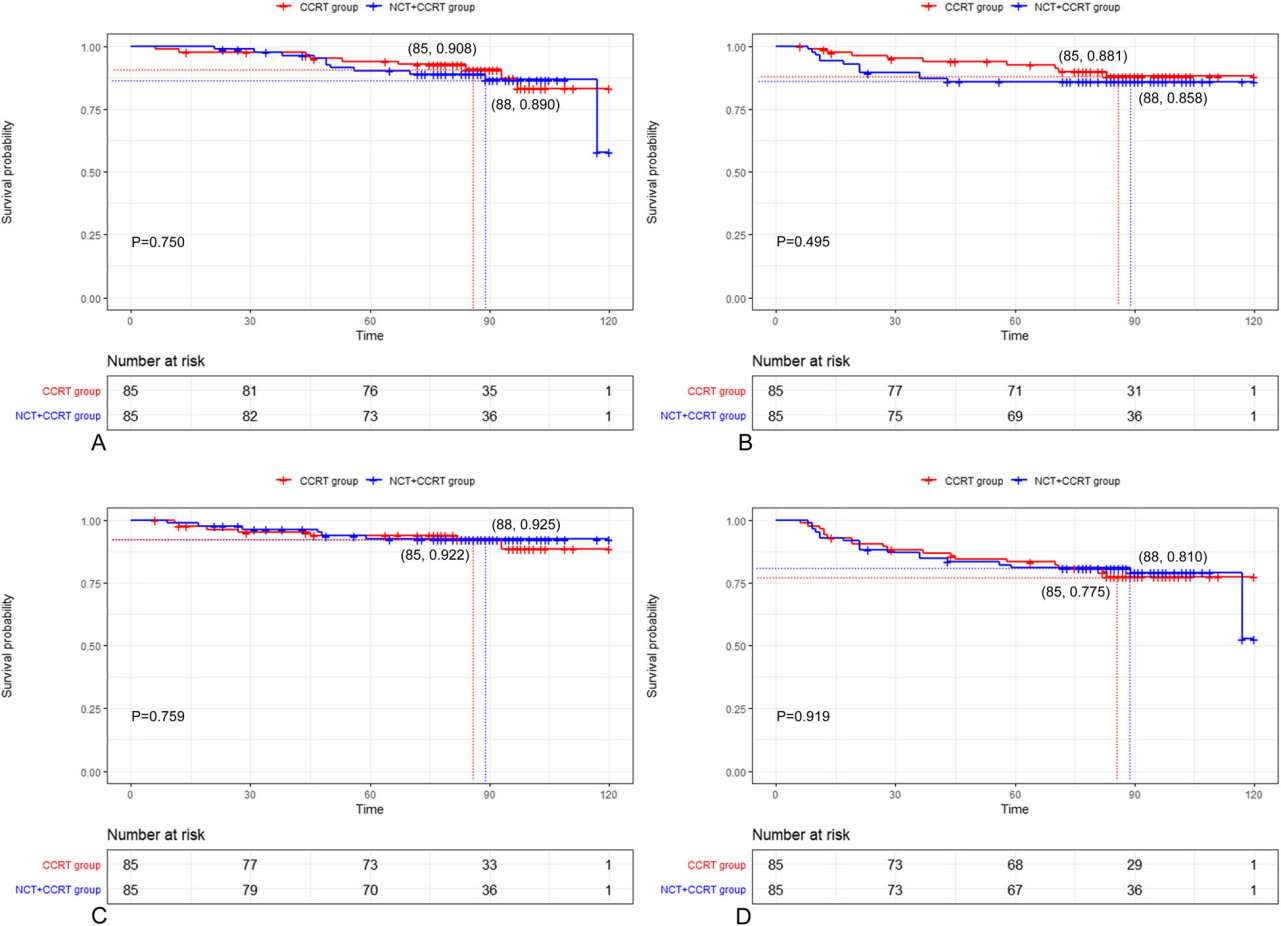
The Kaplan-Meier estimates of overall survival (OS), locoregional recurrence-free survival (LRFS), distant recurrence-free survival (DRFS), and recurrence-free survival (RFS) for NCT + CCRT group and CCRT group in selected 170 patients. The median follow-up in NCT + CCRT group and CCRT group were 88 months and 85 months, respectively. Overall survival (**a**), locoregional recurrence-free survival (**b**), distant recurrence-free survival (**c**), and recurrence-free survival (**d**). *P*-values were calculated by the unadjusted log–rank test. NCT, neoadjuvant chemotherapy; CCRT, concurrent chemoradiotherapy

Follow-up was updated in October 2019 in the entire cohort, with the median follow-up time of 86.5 months (range, 6–120 months). The failure patterns between NCT + CCRT group and CCRT group were concluded in Table 2 with no significant difference. Locoregional recurrence alone occurred in 9 patients in NCT + CCRT group and 8 patients in CCRT group, respectively. Three patients had distant recurrence alone in NCT + CCRT group, while 6 patients in CCRT group had distant recurrence alone. There were 3 of both locoregional and distant recurrence in NCT + CCRT group, and 1 of both locoregional and distant recurrence in CCRT group. At the end of the follow-up, 11 patients died in NCT + CCRT group, while 9 patients died in CCRT group. The survival outcomes between NCT + CCRT group and CCRT group revealed no significant difference in terms of 5-year OS (90.2% vs. 94.0%, *p* = 0.750), LRFS (85.8% vs. 92.6%, *p* = 0.495), DRFS (92.5% vs. 93.9%, *p* = 0.759), and RFS (81.0% vs. 83.3%, *p* = 0.919) (Fig. 2).

**Table 2:** Failure patterns of NCT + CCRT group and CCRT group

**Variables**	**NCT + CCRT group (*N*** **= 85)**	**CCRT group(*N*** **= 85)**	***P*value**
Locoregional recurrence alone	9 (10.6%)	8 (9.4%)	0.486
Distant recurrence alone	3 (3.5%)	6 (7.0%)	0.774
Locoregional and distant recurrence	3 (3.5%)	1 (0.01%)	0.313
Death	11 (12.9%)	9 (10.69%)	0.635

Abbreviations: *NCT* Neoadjuvant chemoradiotherapy; *CCRT* Concurrent chemoradiotherapy

### Univariate and multivariate analysis

Univariate and multivariate analysis were performed to evaluate various prognostic factors (Table 3 and Table 4). Multivariate analysis found that smoking history (hazard ratio [HR] 2.612, 95% confidence interval [CI]: 1.027–6.645 *P* = 0.044) and T classification (HR 2.846, 95% CI: 1.125–7.201, *P* = 0.027) were independent prognostic factors for OS, lymph node diameter (HR 2.617, 95% CI: 1.084–6.318, *P* = 0.032) was independent prognostic factor for LRFS, and positive PLNC was independent prognostic factor for DRFS (HR 4.522, 95% CI: 1.513–13.514, *P* = 0.007), and RFS (HR 2.583, 95% CI: 1.263–5.284, *P* = 0.009).

**Table 3:** Univariate analysis of prognostic factors for 170 patients with stage T3-4N1 NPC

**Variables**	**OS**		**LRFS**		**DRFS**		**RFS**	
	**HR (95% CI)**	**P**	**HR (95% CI)**	**P**	**HR (95% CI)**	**P**	**HR (95% CI)**	**P**
Sex (male vs. female)	0.904 (0.455–1.796)	0.774	0.968 (0.720–1.301)	0.828	0.784 (0.219–2.800)	0.708	0.893 (0.524–1.520)	0.676
Age (years)	1.018 (0.974–1.064)	0.426	0.961 (0.919–1.006)	0.092	1.009 (0.956–1.065)	0.736	1.001 (0.969–1.035)	0.931
Smoking history (yes vs. no)	3.595 (1.465–8.819)	0.005	1.379 (0.535–3.556)	0.506	2.073 (0.676–6.353)	0.202	2.333 (1.192–4.565)	0.013
Alcohol history (yes vs. no)	0.485 (0.064–3.668)	0.483	0.587 (0.079–4.374)	0.603	0.909 (0.118–7.031)	0.927	0.297 (0.040–2.175)	0.232
Family of cancer (yes vs. no)	0.848 (0.308–2.334)	0.749	0.756 (0.277–2.063)	0.585	1.102 (0.339–3.581)	0.871	0.966 (0.466–2.004)	0.927
T classification (T3 vs. T4)	3.997 (1.633–9.782)	0.002	2.008 (0.810–4.980)	0.132	2.548 (0.833–7.800)	0.101	2.158 (1.080–4.313)	0.029
PLNC (negative vs. positive)	3.038 (1.191–7.747)	0.020	1.975 (0.766–5.096)	0.159	4.522 (1.513–13.514)	0.007	3.132 (1.576–6.227)	0.001
Lymph node diameter (mm, maximum, ≤15.0 vs. > 15.0)	2.546 (1.037–6.252)	0.041	2.617 (1.084–6.318)	0.032	2.532 (0.827–7.749)	0.104	2.335 (1.202–4.535)	0.012
Cervical lymph node (yes vs. no)	1.007 (0.377–2.688)	0.989	1.294 (0.474–3.531)	0.615	0.605 (0.198–1.851)	0.378	0.840 (0.416–1.696)	0.627
EBV DNA (copy/ml, < 2000 vs. ≥2000)	1.176 (0.483–2.864)	0.720	1.163 (0.490–2.760)	0.733	3.022 (0.831–10.993)	0.093	1.456 (0.742–2.856)	0.274
HGB (g/L, < 113 vs. 113–151 vs. ≥151)	0.746 (0.305–1.826)	0.521	1.066 (0.465–2.442)	0.880	0.998 (0.342–2.913)	0.997	1.068 (0.565–2.017)	0.840
CRP (g/ml, < 1.0 vs. 1.0–3.0 vs. ≥3.0)	1/366 (0.791–2.359)	0.263	1.204 (0.715–2.027)	0.484	0.644 (0.310–1.337)	0.238	1.215 (0.811–1.820)	0.345
LDH (U/L, < 245 vs. ≥245)	0.045 (0–215.889)	0.473	0.692 (0.093–5.153)	0.719	0.045 (0–882.623)	0.538	0.406 (0.056–2.966)	0.374
NCT (yes vs. no)	1.155 (0.476–2.801)	0.750	1.349 (0.568–3.201)	0.497	0.843 (0.283–2.510)	0.759	0.967 (0.502–1.862)	0.919

Abbreviations: *NPC* Nasopharyngeal carcinoma; *HR* Hazard ratio; *CI* Confidence interval; *NCT* Neoadjuvant chemotherapy; *PLNC* Pretreatment lymph node condition; *EBV* Epstein–Barr virus; *OS* Overall survival; *LRFS* Locoregional recurrence-free survival; *DRFS* Distant recurrence-free survival; *RFS* Recurrence-free survival; *HGB* Hemoglobin; *CRP* C-reactive protein; *LDH* Lactate dehydrogenase

**Table 4:** Multivariate analysis of prognostic factors for 170 patients with stage T3-4N1 NPC

**Variables**	**OS**		**LRFS**		**DRFS**		**RFS**	
	**HR (95% CI)**	**P**	**HR (95% CI)**	**P**	**HR (95% CI)**	**P**	**HR (95% CI)**	**P**
Smoking history (yes vs. no)	2.612 (1.027–6.645)	0.044	–	–	–	–	1.840 (0.913–3.709)	0.088
T classification (T3 vs. T4)	2.846 (1.125–7.201)	0.027	–	–	–	–	1.590 (0.771–3.279)	0.209
PLNC (negative vs. positive)	2.414 (0.900–6.474)	0.080	–	–	4.522 (1.513–13.514)	0.007	2.583 (1.263–5.284)	0.009
Lymph node diameter (mm, maximum, < 15 vs. ≥15)	1.563 (0.597–4.094)	0.363	2.617 (1.084–6.318)	0.032	–	–	1.597 (0.783–3.256)	0.198

Abbreviations: *NPC* Nasopharyngeal carcinoma; *HR* Hazard ratio; *CI* Confidence interval; *PLNC* Pretreatment lymph node condition; *EBV* Epstein–Barr virus; *OS* Overall Survival; *LRFS* Locoregional recurrence-free survival; *DRFS* Distant recurrence-free survival; *RFS* Recurrence-free survival

### Subgroup analysis

Since patients with positive PLNC tended to have poor survival outcomes, we conducted survival analysis to investigate whether NCT was able to improve prognosis among these patients. The baseline characteristics were well balanced between NCT + CCRT group and CCRT group except for family of cancer (*P* = 0.029) (supplementary table). We observed no significant difference between NCT + CCRT group and CCRT group, including 5-year OS (84.8%% vs. 81.3%, *P* = 0.470), LRFS (93.3% vs. 72.2%, *P* = 0.157), DRFS (85.6% vs. 74.6%, *P* = 0.607), and RFS (72.7% vs. 55.6%, *P* = 0.304) (Table 5).

**Table 5:** Survival outcomes in NPC patients with positive PLNC between NCT + CCRT group and CCRT group

**Variables**	**NCT + CCRT group (*N*** **= 18)**	**CCRT group (*N*** **= 15)**	***P*value**
5-year OS	84.8%	81.3%	0.470
5-year LRFS	93.3%	72.2%	0.157
5-year DRFS	85.6%	74.6%	0.607
5-year RFS	72.7%	55.6%	0.304

Abbreviations: *NPC* Nasopharyngeal carcinoma; *NCT* Neoadjuvant chemoradiotherapy; *CCRT* Concurrent chemoradiotherapy; *PLNC* Pretreatment lymph node condition; *OS* Overall survival; *LRFS* Locoregional recurrence-free survival; *DRFS* Distant recurrence-free survival; *RFS* Recurrence-free survival

### Toxicity

Grade 3–4 acute adverse events (AEs) in 170 paired patients were listed in Table 6. The most common Grade 3–4 hematological AEs was leukopenia with 33/85 in NCT + CCRT group and 18/85 in CCRT group, respectively (*P* = 0.012). Higher incidences of neutropenia and anemia were also observed in NCT + CCRT group compared with CCRT group (37.6% vs. 11.8%, *P* < 0.001; and 10.6% vs. 3.5%, *P* = 0.030; respectively). Mucositis was the most frequent non-hematological Grade 3–4 AEs with no significant difference between NCT + CCRT group and CCRT group (21.2% vs. 25.9%, *P* = 0.471). We observed no treatment-related death in both two groups.

**Table 6:** Grade 3–4 acute adverse events in 170 paired patients with stage T3-4N1 NPC

Variables	NCT + CCRT group (*N* = 85)	CCRT group (*N* = 85)	*P* value
Hematological			
Leukopenia	33 (38.8%)	18 (21.2%)	0.012
Neutropenia	32 (37.6%)	10 (11.8%)	< 0.001
Anemia	9 (10.6%)	3 (3.5%)	0.030
Thrombocytopenia	8 (9.4%)	2 (2.4%)	0.120
Non-hematological			
Mucositis	18 (21.2%)	22 (25.9%)	0.471
Xerostomia	1 (1.2%)	0 (0%)	0.317
Dermatitis	1 (1.2%)	1 (1.2%)	1.00
Nausea/ vomiting	8 (9.4%)	3 (3.5%)	0.120
Hepaotoxicity	5 (5.9%)	5 (5.9%)	1.000

**Abbreviations:***NPC* Nasopharyngeal carcinoma; *NCT* Neoadjuvant chemoradiotherapy; *CCRT* Concurrent chemoradiotherapy

## Discussion

NCT followed by CCRT is a popular treatment modality in LANPC in recent years. Theoretically, the application of NCT is likely to shrink tumor volume and eliminate micrometastasis to improve survival outcomes [12]. However, the value of NCT in LANPC is debatable in previous prospective studies. Fountzilas and colleagues [13] compared NCT + CCRT with CCRT in stage IIB-IVB NPC, revealing no significant benefit of NCT in OS or progression-free survival. Another randomized study [14] reported similar results in patients with stage III-IVB NPC. On the other hand, recent clinical trials demonstrated that the use of NCT could improve survival rates in LANPC, especially in terms of distant metastasis. Sun et al. [7] found that three cycles of TPF as additional NCT provided survival benefit in LANPC in 3-year OS (*P* = 0.029), DRFS (*P* = 0.031) and failure-free survival (*P* = 0.034) but LRFS (*P* = 0.12) comparing with CCRT. Zhang et al. [9] explored three cycles of gemcitabine with cisplatin as NCT in LANPC, showing improved 3-year OS (HR: 0.43, 95%CI 0.24–0.77), RFS (HR: 0.51, 95%CI 0.34–0.77), DRFS (HR: 0.43, 95%CI 0.25–0.73), but LRFS (HR: 0.77, 95%CI 0.42–1.41). Moreover, Yang and colleagues [8] reported an OS benefit of NCT (5-year OS of 80.8% in NCT + CCRT group and 76.8% in CCRT group, *P* = 0.040) in LANPC in their updated analysis, and the 5-year DMFS was also improved with additional NCT (82.8% vs. 73.1%, *P* = 0.014). Thus, NCT + CCRT become the main treatment strategy for LANPC in clinical practice.

However, large scale retrospective studies suggested that the distant metastasis rate was 10–15% in patients with stage N1 NPC, while the rate of 30–40% was observed in N2–3 disease [10]. Thus, NPC with N1 stage had relatively low risk of distant metastasis, and the necessary of NCT need further investigation. We collected data from patients with stage T3-4N1 NPC receiving CCRT with or without NCT from 2006 to 2013, and used PSM in 1:1 ratio to balance the baseline characteristics between NCT + CCRT group and CCRT group. The results showed no statistical difference in 5-year DRFS between two groups (92.5% vs. 93.9%, *P* = 0.759), and similar results were found in 5-year OS, LRFS, and RFS. We postulated that the reason of these results might be as follows: (1) with the development of radiotherapy technique, the application of IMRT has greatly increased locoregional control [15], resulting in difficulty for NCT to prolong survival by increasing local control; (2) with relatively low incidence of distant metastasis in N1 disease (10–15%) [10], the value of NCT in reducing distant metastasis and increasing survival might be limited. Thus, although recent clinical trials demonstrated that NCT could improve survival outcomes in LANPC, subgroup analysis of N1 disease presented rare survival benefit [7–9].

Nowadays, the treatment strategy is made based on the AJCC/ UICC stage system [16] and the National Comprehensive Cancer Network (NCCN) guideline [17], which recommended similar treatment plans for patients with stage II-IVA NPC. Oncologists should notice the limitations of these recommendations due to inconsistent benefit of NCT in various subgroups, even in the same clinical trial. The phase III trial conducted by Zhang et al. [9] observed no significant difference in RFS among patients with N1 disease (HR: 1.22, 95%CI 0.63–1.40) between NCT + CCRT group and CCRT group. Moreover, subgroup analysis of N0–1 stage NPC in study performed by Yang et al. [8] revealed no significant difference in terms of disease-free survival (HR: 0.57, 95%CI 0.29–1.14), and OS (HR: 0.64, 95%CI 0.30–1.40). Since previous studies demonstrated that the failure patterns and prognosis differed in patients with different stage disease [18], similar therapy for LANPC might lead to overtreatment and increased costs in low-risk group of LANPC. Therefore, the use of NCT in LANPC with N1 disease needs to be careful in clinical practice, and further evaluations are warrant to verify this issue.

Admittedly, there is still an incidence of around 20% of treatment failures, including locoregional and distant recurrence, in LANPC with stage N1 disease [19]. Thus, identifying high-risk patients and finding individualized treatment strategy are urgent. The current multivariate analysis revealed that the positive PLNC was an adverse prognostic factor of DRFS (*P* = 0.007) and RFS (*P* = 0.009) in stage T3-4N1 NPC. However, we failed to exam out survival benefit of NCT in patients with positive PLNC. We prefer to attribute the negative results to the small sample of the data: only 33/170 patients in the matched cohort had positive PLNC. Therefore, prospective trials are expected to confirm the findings.

On the other hand, we observed significant increased Grade 3–4 hematological AEs in NCT + CCRT group compared with CCRT group (*P* < 0.05). We purposed that higher rates of hematological AEs in NCT + CCRT group might be related to intense chemotherapy. However, in terms of non-hematological Grade 3–4 AEs, including mucositis, xerostomia, and dermatitis, no significant differences were observed between two groups. We postulated that similar incidence of RT related AEs in two groups resulted from advanced RT techniques. With increasing use of IMRT, irradiation dose become higher in target volume and lower in OARs [20]. Moreover, the application of diagnostic imaging such as positron emission tomography/ computed tomography [21] in RT provides opportunities to identify target volume more precise. Therefore, RT related toxicities tend to be decreased and tolerable, which has been reported in treatment of head and neck cancers [22]. In this context, the optimal treatment modality is necessary to be modulated to provide benefit to patients with NPC not only in improving survival outcomes but also in decreasing treatment related AEs.

There were several limitations in this study. Firstly, this is a retrospective study with small sample size. Thus, we failed to identify the value of NCT in patients with high-risk factors such as PLNC. Secondly, the inconsistency of NCT regimens might have impact on the final results. Although there was inevitable selection bias, we used PSM to reduce the heterogeneousness between two treatment groups, and the matched cohorts had balanced baseline characteristics.

## Conclusion

Our results demonstrated that additional NCT on the basis of CCRT in patients with stage T3-4N1 NPC did not provide significant survival benefit but increased acute hematological Grade 3–4 AEs. Positive PLNC was associated with poor survival outcomes, although we failed to identify the efficacy of NCT in this subgroup of patients. Prospective trials are warrant to confirm our findings taking PLNC into consideration.

## Supplementary information

**Additional file 1.** Baseline characteristics of patients with positive PLNC between NCT + CCRT group and CCRT group.

## Data Availability

All data generated or analysed during this study are included in this published article.

## Abbreviations

NPC: Nasopharyngeal carcinoma
LANPC: Locoregionally advanced nasopharyngeal carcinoma
2DCRT: Two-dimentional conventional radiotherapy
IMRT: Intensity-modulated therapy
NCT: Neoadjuvant chemotherapy
CCRT: Concurrent chemoradiotherapy
CCT: Concurrent chemotherapy
OARs: Organs at risk
AJCC/ UICC: American joint committee on cancer/ union for international cancer control
MRI: Magnetic resonance imaging
PLNC: Pretreatment lymph node condition
ICRU: International commission on radiation units and measurements reports
GTV: Gross tumor volume
GTVp: primary gross tumor (including retropharyngeal lymph node metastases)
GTVnd: Cervical lymph node metastasis
CTV1: High-risk clinical target volume
CTV2: Low-risk clinical target volume
PTV: Planning target volume
PF: Cisplatin (80 mg/m2) plus 5-fluorouracil (800 mg/m2/day over 120 h)
TP: Cisplatin (80 mg/m2) plus docetaxel (80 mg/m2)
TPF: Cisplatin (60 mg/m2) plus 5-fluorouracil (600 mg/m2 over 120 h) with docetaxel (60 mg/m2)
OS: Overall survival
LRFS: Locoregional recurrence-free survival
DRFS: Distant recurrence-free survival
RFS: Recurrence-free survival
PSM: Propensity score matching
EBV: Epstein-Barr virus
HGB: Hemoglobin
CRP: C-reactive protein
LDH: Lactate dehydrogenase
HR: Hazard ratio
CI: Confidence interval
NCCN: National Comprehensive Cancer Network
