# Supplementary material for: Can neoadjuvant chemotherapy improve survival in stage T3-4N1 nasopharyngeal carcinoma? A propensity matched analysis
Source: Radiat Oncol. 2020 Jul 2;15:160. doi: 10.1186/s13014-020-01594-4 (PMC7331182; doi:10.1186/s13014-020-01594-4)
Supplement: Supplementary file 1 — Additional file 1. Baseline characteristics of patients with positive PLNC between NCT + CCRT group and CCRT group. [file 13014_2020_1594_MOESM1_ESM.doc]

**Baseline characteristics of patients with positive PLNC between NCT+CCRT group and CCRT group**

| **Variables** | **NCT followed by CCRT group （18）** | **CCRT group**  **（15）** | **P value** |
| --- | --- | --- | --- |
| Sex  Male  Female | 5 (27.8%)  13 (72.2%) | 6 (40.0%)  9 (60.0%) | 0.707 |
| Age (years) | Median: 41,  range: 23-73 | Median: 42,  range: 28-62 | 0.882 |
| Smoking history  No  Yes | 12 (66.7%)  6 (33.3%) | 11 (73.3%)  4 (26.7%) | 0.683 |
| Alcohol history  No  Yes | 18 (100%)  0 (0%) | 14 (93.3%)  1 (6.7%) | 0.273 |
| Family of cancer  No  Yes | 14 (76.5%)  20 (23.5%) | 6 (40.0%)  9 (60.0%) | 0.029 |
| T classification  T3  T4 | 67 (77.8%)  4 (22.2%) | 10 (66.7%)  5 (33.3%) | 0.273 |
| Lymph node diameter (mm, maximum) | Median: 21.5,  range: 10-36 | Median: 18,  range: 5-41 | 0.447 |
| Cervical lymph node  No  Yes | 2 (11.1%)  16 (88.9%) | 1 (6.7%)  14 (93.3%) | 0.663 |
| EBV DNA (copy/ml)  ≤2000  >2000 | 5 (27.8%)  13 (72.2%) | 5 (33.3%)  10 (66.7%) | 0.733 |
| HGB, g/L  <113  113-151  ≥151 | 1 (5.6%)  11 (61.1%)  6 (33.3%) | 0 (0%)  13 (86.7%)  2 (13.3%) | 0.339 |
| CRP, g/ml  <1.0  1.0-3.0  ≥3.0 | 6 (33.3%)  3 (16.7%)  9 (50.0%) | 7 (46.7%)  3 (20.0%)  5 (33.3%) | 0.349 |
| LDH, U/L  <245  ≥245 | 18 (100%)  0 (0%) | 15 (100%)  0 (0%) | 1.000 |

**Abbreviations:** NPC, nasopharyngeal carcinoma; NCT, neoadjuvant chemoradiotherapy; CCRT, concurrent chemoradiotherapy; PLNC, pretreatment lymph node condition; EBV, Epstein–Barr virus; HGB, hemoglobin; CRP, C-reactive protein; LDH, lactate dehydrogenase.
